# Continuous Glucose Monitoring in the Management of Congenital Hyperinsulinism: A National User-satisfaction Survey, UK

**DOI:** 10.1210/clinem/dgaf313

**Published:** 2025-05-26

**Authors:** Helen Couch, Andrew Pearson, Neha Malhotra, Michael Ferguson, George Couch, Clare Gilbert, Kate Morgan, Sarah Mann, Kelly Cassidy, Sarah Worthington, Elaine O'Shea, Maria SalomonEstebanez, Chris Worth, Senthil Senniappan, Indraneel Banerjee, Antonia Dastamani

**Affiliations:** Department of Paediatrics, Cambridge University Hospitals NHS Foundation Trust, Cambridge, CB2 0QQ, UK; Quality and Safety Team, Great Ormond Street Hospital for Children NHS Foundation Trust, London, WC1N 3JH, UK; Department of Paediatrics, Basildon University Hospital, Essex, SS16 5NL, UK; Your Path Solutions, Toronto, ON, M6S2Y1, Canada; Department of Anaesthetics, Cambridge University Hospitals NHS Foundation Trust, Cambridge, CB2 0QQ, UK; Department of Oncology, Cancer Research UK – Cambridge Institute, Cambridge, CB2 0RE, UK; Department of Paediatric Endocrinology and Diabetes, Great Ormond Street Hospital for Children NHS Foundation Trust, London, WC1N 3JH, UK; Department of Paediatric Endocrinology and Diabetes, Great Ormond Street Hospital for Children NHS Foundation Trust, London, WC1N 3JH, UK; Department of Paediatric Endocrinology and Diabetes, Great Ormond Street Hospital for Children NHS Foundation Trust, London, WC1N 3JH, UK; Department of Paediatric Endocrinology, Alder Hey Children’s Hospital, Liverpool, L14 5AB, UK; Department of Paediatric Endocrinology, Royal Manchester Children’s Hospital, Manchester, M13 9WL, UK; Department of Paediatric Endocrinology, Royal Manchester Children’s Hospital, Manchester, M13 9WL, UK; Department of Paediatric Endocrinology, Royal Manchester Children’s Hospital, Manchester, M13 9WL, UK; Department of Paediatric Endocrinology, Royal Manchester Children’s Hospital, Manchester, M13 9WL, UK; Department of Paediatric Endocrinology, Alder Hey Children’s Hospital, Liverpool, L14 5AB, UK; Department of Paediatric Endocrinology, Royal Manchester Children’s Hospital, Manchester, M13 9WL, UK; Department of Paediatric Endocrinology and Diabetes, Great Ormond Street Hospital for Children NHS Foundation Trust, London, WC1N 3JH, UK

**Keywords:** congenital hyperinsulinism, CHI, hypoglycemia, continuous glucose monitoring, CGM, experiences, satisfaction

## Abstract

**Context:**

Congenital hyperinsulinism (CHI) causes severe and recurrent hypoglycemia with a 33% to 50% risk of neurodisability necessitating rigorous glucose monitoring. Continuous glucose monitoring (CGM) is now widely used in CHI with limited data on user-reported benefits. There are no validated instruments available to assess the impact of CGM use on quality of life (QOL) in CHI.

**Objective:**

To evaluate CGM user satisfaction of patients with CHI and their carers.

**Design:**

Modified CGM-satisfaction (CGM-SAT) and glucose monitoring surveys (GMS) were distributed electronically to CHI families using CGM.

**Setting:**

CHI highly specialized services, UK, May to August 2023.

**Patients or Other Participants:**

Parents (n = 86) and teachers (n = 15) of patients with CHI using CGM (0-18 years old) and patients themselves if ≥7 years old (n = 20).

**Main Outcome Measures:**

User-reported ease of CGM handling, influence of CGM on CHI management and QOL, and desire for continued use of CGM.

**Results:**

Most respondents reacted positively to statements regarding: CGM device handling (58% agreed or strongly agreed), influence on CHI management (70%), QOL (75%), and continued use (86%). Satisfaction with CGM was positively correlated with duration of use (r = 0.40, *P* < 0.001). Eighty-six percent of users reported checking the CGM 1 to 5 times per hour. Users reported perceived improvements in safety, hypoglycemia detection, freedom, and independence, despite concerns with accuracy and device range.

**Conclusion:**

Patients with CHI and their carers reported that they feel safer and perceive benefits from CGM in all aspects of living with CHI.

Congenital hyperinsulinism (CHI) is a rare condition characterized by severe and persistent hypoglycemia due to dysregulated insulin secretion from pancreatic β-cells (1) that represents a substantial burden to affected children, their families, and the National Health Service (NHS) (∼£3.5 million annually) (2, 3). Preventing neurocognitive impairment from recurrent hypoglycemia requires strict, often mechanically assisted feeding regimens and carbohydrate supplementation (2, 4). Recurrent hypoglycemia alongside these high-frequency interventions adversely impact a child's normal development, social opportunities, and interactions (3).

CHI families report significant challenges, including fear of hypoglycemia, anxiety about brain injury, and chronic sleep disruption due to overnight monitoring and feeding (5). The Parent Quality of Life Survey 2021 by The HI Global Registry identified a significant proportion of parents reporting poor mental (67%) and physical (48%) health (6). When compared with the relative proportions of UK adults requiring mental health treatment (13%) and reporting poor physical health (6-8%), these figures highlight the burden of chronic health conditions on parents (7-9).

Continuous glucose monitoring (CGM) provides real-time feedback on glucose levels and retrospective 24-hour glucose profiles, offering several significant advantages over self-monitoring blood glucose (SMBG), the current standard of care. Real-time CGM information promotes awareness of current glucose levels alongside recording both short- and long-term trends (10). There is currently no published evidence to demonstrate the efficacy of CGM as a standalone tool in reducing hypoglycemia for children with CHI (4, 11, 12).

Technological advancements have revolutionized blood glucose management for children and young people with diabetes (CYPD) and their families (13, 14), for whom CGM is now a standard of care in the UK (15, 16). This approval followed initial hesitancy from many clinicians due to a lack of efficacy data, despite positive patient-reported outcomes (17). Key to the adoption of CGM for CYPD was demonstrating a reduction in hemoglobin A1c; however, there is no equivalent biomarker to demonstrate a reduction in the severe episodic hypoglycemia seen in CHI.

National surveys by the British Society for Paediatric Endocrinology and Diabetes and the Children's Hyperinsulinism Charity have called for widening CGM access to patients with recurrent hypoglycemia (18). At the time of our study, access to CGM for patients with CHI outside of clinical trials was primarily dependent upon independent funding requests to integrated care boards or self-funding (3, 4, 18).

CGM alerts inform the user when their interstitial glucose is falling rapidly or moving into their personalized hypoglycemic range. The potential benefit from such alerts for patients and carers in the community is clear: to prompt additional targeted SMBG checks, to ensure that medications and feeds have been delivered as per the patients care plan, to provide preemptive snacks or hypotreatment where appropriate, and to consider factors that may be impacting glucose levels such as physical activity and environment. Long-term trends allow retrospective analysis, which can be helpful for families, particularly when supported by their endocrinologist, to identify vulnerable times and adjust regimens to minimize the risk of hypoglycemia (11, 19).

The UK consensus statement on the management of CHI acknowledges a role for CGM in outpatient CHI management by mitigating the risk of life-threatening hypoglycemia overnight (4), a time of increased vulnerability for children with CHI (20), in particular for those dependent upon feeding pumps. Interruption of continual enteral glucose delivery may occur either through technical fault or caregiver error, resulting in severe and prolonged episodes of hypoglycemia that can lead to neurodisability and death (18, 21).

The CGM-satisfaction survey (CGM-SAT) and glucose monitoring survey (GMS) have been assessed to be reliable and valid measures of patient-reported CGM outcomes in type 1 diabetes (T1D) (19). Previous use of the CGM-SAT surveys in T1D has highlighted the positive impact of CGM on quality of life for children and adolescents (14, 19) and emphasized the need to integrate patient-reported satisfaction into clinical practice to enhance patient and carer well-being (13).

Studies thus far have demonstrated a concerning lack of sensitivity for detecting hypoglycemia in CHI (22). A cohort of patients provided CGM for a prospective study had a 50% discontinuation rate, citing pain and inaccuracy as significant problems (23). Despite this, patients and families have consistently reported benefits from CGM, including improved quality of life, identification of new episodes of hypoglycemia, improved understanding of glucose trends, and improved management of CHI (22). A previous study conducted interviews with 9 families, revealing that while device range, accuracy, and some aspects of alarms were issues, CGM allowed them to identify more hypoglycemic episodes than finger-prick tests, provided reassurance, and allowed modification of daily routines to help CHI management (24).

This study aims to build on this data by presenting the benefits and problems with using CGM as an adjunct to SMBG in managing CHI and identifying the outcomes and improvements most valuable to users, as perceived by this national cohort of active CGM users.

## Methods

Given the absence of a prevalidated survey for patients with CHI, we implemented a systematic approach to enhance the validity and robustness of the CGM-SAT and GMS surveys. A multidisciplinary expert panel was convened, comprising pediatric endocrinologists, hypoglycemia nurse specialists, a psychologist, and a clinical audit manager from across the 3 CHI highly specialized services within the NHS in England, at Great Ormond Street Hospital, Royal Manchester Children's Hospital, and Alder Hey Children's Hospital.

These experts, possessing extensive experience in CHI management, collaborated virtually over 6 months, concluding in May 2023, to develop qualitative surveys informed by user feedback surveys of continuous and conventional glucose monitoring in CYPD (19). This produced 4 surveys: the CGM-SAT survey for parents (52-item questionnaire), for patients (42-item questionnaire), and for teachers (16-item questionnaire) and the GMS survey for parents or patients (13-item questionnaire). These are included as Supplementary Material (25).

The study protocol was reviewed and approved by the Great Ormond Street Hospital (GOSH) Clinical Audit Team who found that it met the national definition of a service evaluation, in line with the criteria outlined by the NHS Health Research Authority. Therefore, this study did not require review by the GOSH Research Ethics Committee, as it discussed feedback on the impact and effectiveness of an approved existing intervention. All questionnaire responses were anonymized, with no personally identifiable information collected. Informed consent from the participants was gained by recruiting clinicians, and participants were advised about the use of their data for publication purposes.

To refine the survey and identify potential limitations, a pilot test was conducted with a CHI-affected family of 1 patient over 7 years of age and their caregivers from an international setting. The feedback obtained facilitated the identification and resolution of potential issues, ensuring the survey's clarity and relevance. Additionally, free text responses were incorporated to capture qualitative insights from respondents, providing contextual depth and highlighting areas for further refinement.

The CGM-SAT surveys were designed to measure the impact of using CGM on CHI management; family relationships; and user emotional, behavioral, and cognitive satisfaction. They included both positive and negative statements regarding (1) use of the CGM device, including confidence, helpfulness, accuracy, difficulties, side effects, and device range; (2) perception of CHI management, including confidence, awareness and control, frequency of SMBG checks and hypoglycemic episodes, and identification of hypoglycemia; (3) quality of life as measured by fears, perceived safety, freedom, interruptions, emotions, and interpersonal issues; and (4) desire for continued use of CGM. Respondents rated the level of agreement with each statement on a 5-point Likert scale (1 = strongly disagree to 5 = strongly agree). Questionnaires were edited to only include questions relevant to each respondent group (patients, parents, and teachers). Likert scores for statements with negative sentiment toward CGM were inverted during data analysis, so that higher scores always reflected more favorable impact and satisfaction with CGM.

The GMS (19) is a 13-item scale where families rated their satisfaction with, and the therapeutic impact of, their glucose monitoring system before (SMBG alone) and after (SMBG alongside CGM) using CGM. Each item asks the user to evaluate a statement with respect to “Was this a problem before using a CGM?” and “How has it changed after using a CGM?” Problems were reported on a 4-point Likert scale (1 = a lot to 4 = not at all). Changes were also reported on a 4-point Likert scale (1 = much worse to 4 = a bit better). Higher scores reflected less severe problems prior to CGM and more positive change with CGM.

Families with a child (≤18 years old) with CHI who were using CGM at the time of recruitment were eligible to take part in the study. Surveys were distributed electronically by endocrinologists working within the CHI highly specialized services. Children with CHI (aged 7-18 years) were invited to share their own thoughts using the CGM-SAT survey for patients. If the child with CHI was accessing education, the family was invited to share a link to the CGM-SAT for teachers with their child's teacher. A single reminder email was sent to all participants. Responses were collected over a 16-week period between May 5, 2023, and August 25, 2023.

Data analysis was performed in R v4.3.1, utilizing the likert v1.3.5 and ltm v1.2-0 packages. The change in mean response score of parents and patients was modeled using multiple linear regression with assumptions checked using residual, Q-Q, location-scale, and Cook's distance plots. Internal reliability for patients', parents', and teachers' CGM-SAT and the GMS surveys was calculated using Cronbach's α coefficients. Parent-patient agreement was assessed using the Spearman rank-order correlation coefficient. Significance between subgroups was examined using Kruskal-Wallis tests followed up, if appropriate, with pairwise Wilcoxon tests with Bonferroni correction.

## Results

### Demographics

One hundred sixty-seven surveys were returned of the 282 issued (59.2%). Response rate varied by institution and survey (Table 1), with a majority of responses from Great Ormond Street Hospital (n = 131, response rate = 63.6%). CGM-SAT survey responses of both parents (n = 86) and patients (n = 20) showed excellent parent-patient correlation (0.82), and there was strong internal consistency (α coefficients 0.87-0.95). The GMS survey demonstrated acceptable internal consistency, with an α coefficient of 0.73.

**Table 1.** Descriptive statistics of the survey data

		Parent CGM-SAT	Patient CGM-SAT	Teacher CGM-SAT	GMS
Responses	Response count (surveys issued)	86 (130)	20 (37)	16 (36)	45 (79)
	Percentage response return	66.2	54.1	44.4	57.0
	Great Ormond Street Hospital (surveys issued)	60 (94)	19 (30)	15 (35)	37 (47)
	Alder Hey Children's Hospital (surveys issued)	12 (12)	1 (1)	1 (1)	8 (8)
	Royal Manchester Children's Hospital (surveys issued)	14 (24)	0 (6)	0 (0)	0 (24)
Patient age (years)	Median	7	9.5	8.5	
	Interquartile range	6.5	6	4	
	Minimum	1	7	2	
	Maximum	18	17	17	
Duration CGM use (months)	Median	15	24		
	Interquartile range	19.25	37.25		
	Minimum	1	2		
	Maximum	76	66		
Item score	Mean ± SD	3.9 ± 1.1	3.8 ± 1.2	4.0 ± 1.0	
	Median	4.0	4.0	4.0	
	α coefficient	0.95	0.87	0.95	0.73
	Parent-patient correlation	0.82			

There were 167 responses from 282 issued surveys, representing a response rate of 59%. Response rates are in the format: number of responses received (number of surveys issued). Item scores are presented with mean score ± SD, Cronbach's α coefficient for internal reliability within each respondent group, and parent-patient agreement Spearman rank-order correlation coefficient.

Abbreviations: CGM, continuous glucose monitoring; GMS, glucose monitoring survey; SAT, satisfaction.

### CGM Satisfaction Survey

**Figure 1.**
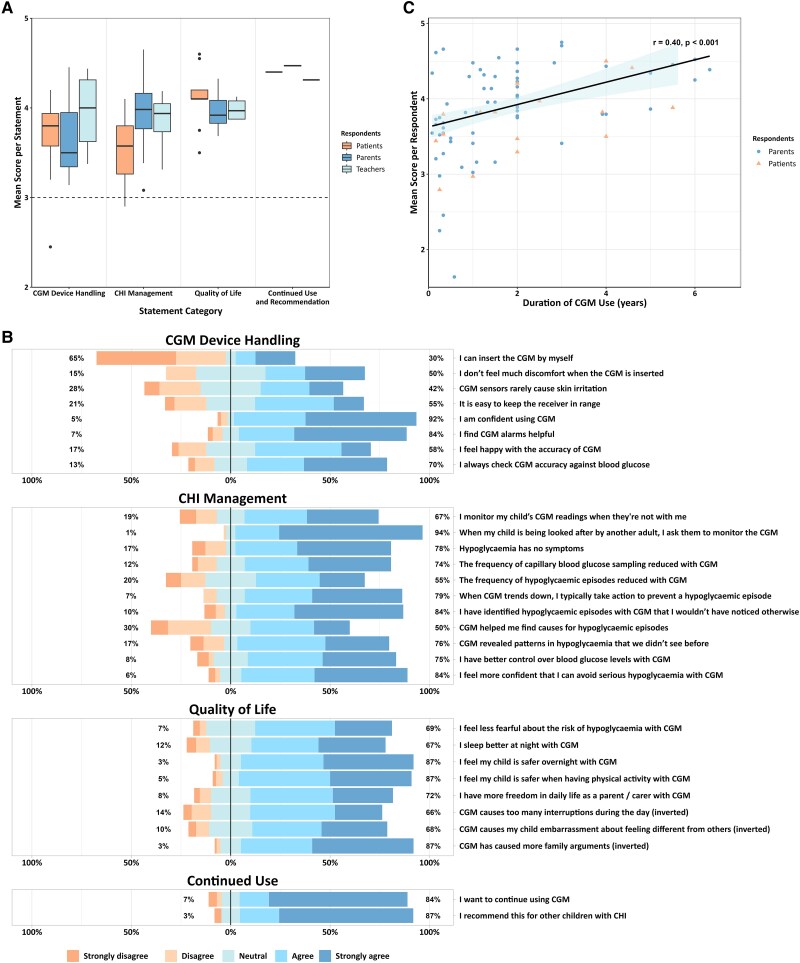
(A) CGM-SAT survey: boxplot of the mean score of each statement against statement category, colored by respondent group. A score of 5 reflected a strongly positive response, 3 was neutral, and 1 was a strongly negative response. There were no statistically significant differences in the mean scores per statement between respondent groups within each statement category. (B) Selected representative CGM-SAT survey results divided by statement domain. The complete results divided by respondent group are included in Supplementary Fig. S1 (25). Statements with negative connotations have had their answers inverted so that “strongly agree” responses all relay positivity about CGM. (C) Scatter plot of mean score per respondent (average respondent score across all CGM-SAT statements) vs duration of CGM use (years), colored by respondent group. The regression line represents the additive effects of duration of CGM use and respondent group, as the interaction term was not significant (*P* = .83). The shaded area indicates the 95% confidence interval. Abbreviations: CGM, continuous glucose monitoring; SAT, satisfaction.

Responses were positive toward CGM use, with no significant differences between respondent groups within each statement category [Fig. 1A-1B, Supplementary Fig. S1 (25)].

1. CGM device handling—The majority of respondents reported confidence using CGM (92%), found CGM alarms helpful (84%), and felt happy with device accuracy (58%). Seventy percent reported that they always used their glucometer to confirm the accuracy of CGM readings. A minority of patients (30%) could independently insert their CGM; however, only 17% of parents reported problems with insertion. A small proportion of respondents indicated discomfort or pain on CGM insertion (15%) and difficulties with device range (21%).
2. CHI management—Patients (60%) and parents (82%) agreed that CHI causes asymptomatic hypoglycemia. Respondents agreed that CGM allowed identification of hypoglycemic episodes that would have been missed with SMBG checks alone (84%); episodes are typically confirmed by SMBG check demonstrating ≤3.5 mmol/L. Respondents also reported reduced frequency of SMBG checks (74%) and improved control over blood glucose levels (75%) and that trend information enabled preventative actions when glucose levels were falling (79%). Almost all parents (94%) asked other adults to monitor the CGM when caring for their child, and 67% of parents continued to monitor their child's CGM when they were apart. Teachers reported that CGM enabled greater awareness of the child's blood glucose (62%). While patients tended to agree that CGM use improved school performance (50% agreed, 20% disagreed), the majority of parents (71%) and teachers (50%) responded neutrally to this statement.
3. Quality of life*—*Respondents agreed that CGM made them feel less fearful about the risk of hypoglycemia (69%) and safer both during episodes of physical activity (87%) and overnight (87%). Parents and carers reported that CGM allowed them more freedom in their daily lives (72%) and improved their sleep (69%). No patients indicated that wearing CGM caused embarrassment, although 12% of parents reported that it did. The majority of respondents disagreed that CGM caused too many interruptions (66%) or family arguments (87%).
4. Continued use*—*Respondents wished to continue using CGM (84%) and would recommend CGM for other CHI patients (87%).

One hundred four respondents (84 parents and 20 patients) provided their duration of CGM use. Multiple linear regression was used to test if duration of CGM use, adjusted for respondent group, predicted mean score per respondent for the CGM-SAT survey. The interaction term between duration and respondent group was nonsignificant (*P* = .83); therefore, an additive model was used with the fitted regression equation: mean = 3.66 + 0.159(duration) – 0.25 (respondent group). Model assumptions were verified using residual plots. Duration of CGM use was a positive predictor of mean score (β = .159, 95% confidence interval: 0.008 to 0.019, *P* < .001), while the respondent group showed a marginal association (β = −.25, *P* = .050). The model explained 18% of the variance in mean scores [adjusted R^2^ = 0.181, F(2, 101) = 12.4], with a moderate correlation between predicted and observed values (r = 0.40, *P* < .001).

**Figure 2.**
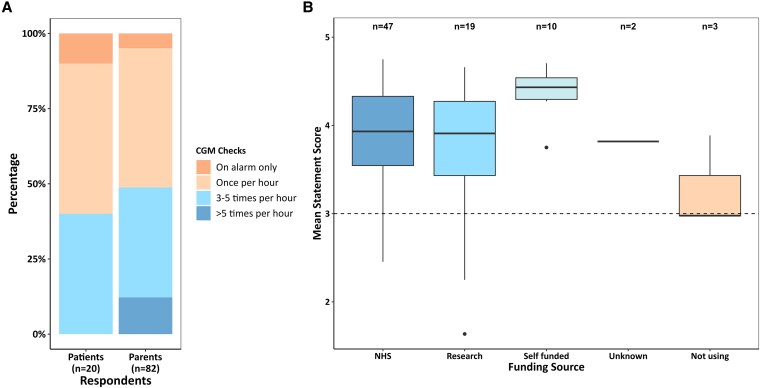
(A) Stacked bar chart showing frequency of checking CGM on a 4-point Likert scale by respondent group. (B) Boxplot of mean score across all survey statements against self-reported CGM funding source. “n” is the total number of respondents citing each funding source. There were no statistically significant differences in the mean scores between funding sources. Abbreviation: CGM, continuous glucose monitoring.

The majority of patients and parents reported checking CGM readings at least once per hour, and 50% of parents checked at least 3 times per hour. A small minority reported checking CGM readings only in response to alarms (Fig. 2A).

Regarding CGM funding (Fig. 2B), 78 of 81 respondents were using CGM at the time of survey completion. Sixty percent of CGMs were funded by the NHS, 23% through research trials, and 12% self-funded. No respondents used insurance-based funding. Of those self-funding, 2 reported that their NHS funding had been withdrawn, and 2 reported that their access to CGM through a research trial had ended. Of those not using CGM, 1 respondent reported their NHS funding had been withdrawn. There were no significant differences in mean scores between funding groups.

### Glucose Monitoring Survey

A transcription error upon building the electronic version of the GMS meant that respondents were unable to indicate whether CGM had impacted an aspect of care by making it “much better.” The remaining positive response, “a bit better,” was renamed “better” to aid interpretation.

**Figure 3.**
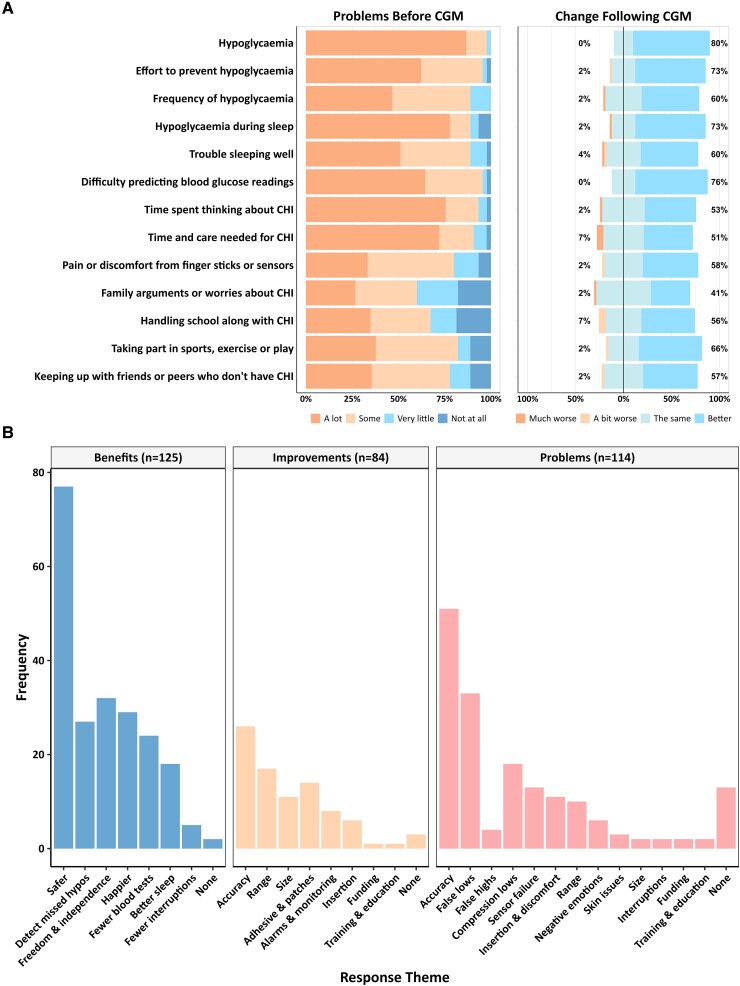
(A) GMS survey responses on 4-point Likert scales. (B) Bar chart showing the frequency of identified themes arising in free text comments to CGM-SAT and GMS surveys. “n” is the total number of comments in each category. Abbreviations: CGM, continuous glucose monitoring; GMS, glucose monitoring survey; SAT, satisfaction.

The majority of respondents (n = 45) agreed that they experienced all problems listed “some” or “a lot” of the time before using CGM (Fig. 3A). Respondents indicated that the frequency of (60%) and the effort required to prevent (73%) episodes of hypoglycemia improved with CGM. Nocturnal hypoglycemia (73%) and sleep quality (60%) are significant problems for families, both of which were better with CGM. Families reported that blood glucose was easier to predict with CGM (76%), and the burden of care had reduced (51%). The majority (58%) felt that overall discomfort from finger sticks or sensors improved with CGM.

Regarding the patients' daily lives, respondents indicated that CGM improved ease of managing school alongside their condition (56%), and in engagement with sports, exercise, or play (66%), and allowed patients to keep up with their peers (75%).

### Free Text Responses

The surveys included the opportunity for free text feedback in 3 areas: “what have been the benefits of having CGM” (n = 125 comments), “what could be further improved about CGM?” (n = 84), and “what have been the problems with having CGM?” (n = 114) (Fig. 3B). The most common sentiments regarding the benefits of CGM referenced safety, happiness, freedom, and independence. Carers and families desire improvements in accuracy, range, and convenience with smaller wearable receivers. Respondents highlighted problems with accuracy, in particular false hypoglycemic readings and sensor failures.

## Discussion

This national patient survey was developed by UK CHI specialists following an initial study of patient and family experience of CGM (24) to explore the CGM user experience and satisfaction of patients with CHI, their parents, and their teachers. The CGM-SAT survey is a patient satisfaction tool validated in diabetes, and our results showed that when adapting for CHI, the surveys maintained high internal consistency (19).

The total number of patients in the UK with CHI and, of those, the proportion using CGM at the time of the survey is unknown. Our survey achieved a high response rate; however, this varied widely between the specialist centers, which may indicate selection bias. The preponderance of GOSH patient families surveyed may reflect that this center has more patients accessing CGM than others but regardless skews the collected data to reflect the opinions of families under the care of this center.

During the study period, access to CGM through the NHS for patients with CHI was secured through independent funding requests negotiated with each child's local care board, leading to significant inequality in access (4, 18). The CHI centers have subsequently acquired improved funding through the Specialised Commissioning NHS England Committee for a limited cohort of patients with CHI dependent upon overnight enteral feeding pumps, widening access (21). We did not collect data on household income or expenditure related to CGM use; however, this may be an important factor impacting satisfaction and could be included in future work.

Our data demonstrate that CHI patients and families using CGM do find it a helpful tool that they would recommend to others. However, many factors may influence engagement with CGM technology and the perception of benefit to burden that CGM provides (26). These include the timing of CGM introduction during the patient journey, severity of disease including the frequency of overnight interventions, individual parent/carer and child ages, personalities and temperaments, specific devices offered, provision of specialist education and support, and the opinions of patients' managing clinicians. Differences between patient and family groups who engage well with or discontinue CGM use should be further explored to allow targeted provision of appropriate support.

The increase in satisfaction with duration of use may be an instance of reverse causality, where higher satisfaction leads to longer duration of use. However, even respondents who had been using CGM for a short period of time had generally positive mean scores. There are multiple reasons why increased duration of use may lead to improved user satisfaction, including the learning curve for effectively utilizing the technology, the ability to identify important trends, optimizing use for the individual (eg, sensor placement, avoiding compression lows, managing device range), and familiarity with alarms. Future surveys could explore the details of this correlation to identify ways of improving satisfaction earlier.

This study is of CGM users and therefore has a positive sampling bias due to a lack of data on the experiences of families who do not use CGM or had discontinued CGM. Discontinuation rates among families who are provided CGM in research trials are markedly high. One study showed a 33% discontinuation rate after 12 weeks (24), and another found 50% of CHI families chose to discontinue CGM despite free-of-charge provision of consumables (23). In the latter study, 71% of participants were CGM-naïve, and this group had the highest dropout rate. Despite the majority of parents citing the benefits of CGM on CHI management, only 41% of families reported that the positives outweighed the negatives (pain and sensor accuracy). In another abstract (27), a series of interviews with families who had discontinued CGM identified pain, accuracy, and device set-up as the main complaints. It is important that future studies target this group to better understand how they can be served by CGM technology.

Data regarding the CGM device used were not collected; we are therefore unable to assess whether different devices affect user satisfaction. No data was collected on the provision of CGM education, specialist support, and interpretation provided to families or how this varied between CHI centers; we acknowledge that this may skew satisfaction with CGM (18, 28). Clinicians have an instrumental role in supporting patient decision-making; their opinion of the benefits and limitations of this technology for their patient may influence parental engagement. Future studies are encouraged to collect data on the approaches of different centers on supporting CHI families with CGM technology.

Some survey statements contained ambiguity. The statement “CGM sensors rarely cause skin irritation” had a fairly even spread of responses, perhaps because it was unclear if it was asking whether skin irritation was *rare* or if CGM use *causes* skin irritation. Studies have found the management of 20% to 90% of children with T1D is impacted by allergic and irritant contact dermatitis to adhesives and components of wearable medical devices (29, 30). Future research should clarify the impact of adverse skin reactions on CGM use in the CHI population, who are unique in that they are increasingly likely to encounter CGM devices from the neonatal period (11, 31, 32). The discrepancy between parent and teacher and child responses regarding school performance may also be affected by ambiguity, where adults may associate school performance with academic grades but children may interpret this statement more broadly, eg, that they feel more engaged or better able to concentrate during the school day, with less time consumed by their medical care. More research on the impact of CGM in the classroom would be helpful to better understand this trend.

In the free text comments, some respondents expressed frustration about receiver range, eg, “My son plays a lot of sports eg, football so is frequently out of range.” However, in the survey, no patients, and only 26% of parents, reported difficulty with keeping the receiver within range. “No needle (insertion)” was suggested as an improvement, despite a minority indicating that discomfort on insertion was a problem. Pain and discomfort appeared to be much less of an issue for our respondents compared to other studies (23). In fact, respondents indicated that pain was better following use of CGM, perhaps reflecting the reduced burden of SMBG checks. While many patients disagreed that they could “insert the CGM by [themselves]”, this is expected given the median age of 9.5 years of children responding independently.

Hypoglycemic unawareness is an important aspect of CHI, as episodes of hypoglycemia are often unpredictable, sudden, and severe (33). The strong agreement of both carers and patients with the statement “hypoglycemia has no symptoms” highlights a key challenge in managing CHI. Interestingly, fewer patients agreed with this statement than parents; this may indicate a disparity between parents' perception of the signs of hypoglycemia and patients' experience of the symptoms of hypoglycemia. When a child does not manifest any external signs of hypoglycemia or both the child and caregiver are unable to recognize the signs or symptoms, scheduled SMBG checks alongside responsive symptoms-based checks are likely to underdetect hypoglycemic episodes (18). This was also noted in the free text comments, eg, “Unexpected hypoglycaemic episodes would effectively be impossible for us to catch with her glucometer.”

The statement “I always check CGM accuracy against blood glucose” lacked clarity in the form of a timeframe, eg, checking the accuracy of each new sensor daily with routine scheduled SMBG checks or following hypoglycemic alerts. Parents' and teachers' responses were more positive to this statement than patients', it may be that knowledge of what they *should* do has influenced their responses or that patients are less compliant with this important safety measure. “Being more accurate” was identified in our survey as the top improvement users want. A recent analysis of paired SMBG and CGM data demonstrated a *higher* average reading of CGM compared to SMBG (23). This contrasts with our findings and previous studies (22, 24, 27), where false and compression lows were reported more frequently than false highs. One explanation for this may be that falsely low CGM readings generate alarms prompting confirmatory SMBG checks, whereas falsely high CGM readings may be in the normal range and therefore only be detected with scheduled SMBG checks. CHI patients and caregivers using CGM are strongly encouraged to maintain regular SMBG checks alongside CGM use and to confirm hypoglycemia with SMBG [usually ≤ 3.5 mmol/L in the UK (4)].

Studies comparing CGM with paired SMBG readings have demonstrated low sensitivity for hypoglycemia (∼30-55%), with a high specificity (>95%) (22, 23). This paired data indicates many hypoglycemic episodes are missed by CGM, suggesting that CGM may provide an element of false reassurance and reinforcing the importance of continuing regular SMBG checks alongside CGM use. However, our users reported that CGM allowed detection of hypoglycemic episodes that would have been missed with scheduled SMBG checks alone. This is subjective, as we have no data for the actual number of hypoglycemic episodes within this study, and yet this sentiment may be accurate, as CGM may still detect more hypoglycemic events than scheduled and symptomatic SMBG checks despite not detecting all hypoglycemic events. The period of greatest risk of hypoglycemia for patients with CHI has been demonstrated to occur in the early hours of the morning, peaking between 0400 and 0700, yet scheduled overnight SMBG checks are performed infrequently (23, 34). Even using CGM with 50% sensitivity may alert carers to undertake SMBG checks to confirm and treat approximately half of these episodes, reducing the risk of neurological damage. This was described in a recent abstract on the inpatient utility of CGM (35) that showed CGM identified a significant burden of hypoglycemia (209 hours), which would not be detected through scheduled SMBG checks alone over 5 inpatient admissions (146 inpatient days). While it is both important and desirable that manufacturers prioritize improving CGM accuracy to protect users from undetected hypoglycemic events, this does not invalidate the utility of CGM as an adjunct method to support the detection of asymptomatic hypoglycemia between scheduled SMBG checks.

Responses to the statement “the frequency of hypoglycemic episodes reduced with CGM” were more muted, with 25% responding neutrally and 20% of respondents disagreeing. As respondents reported that CGM allowed detection of hypoglycemic episodes that would otherwise have been missed, many patients may have experienced an increase in identified hypoglycemic episodes. Through rapid detection and treatment of hypoglycemia, it may be expected that patients using CGM would experience a reduction in time below range rather than discrete hypoglycemic episodes. However, CGM has not yet been demonstrated to reduce the frequency of hypoglycemic episodes or time below range for patients with CHI (23). Users reported utility from CGM through monitoring trends and responding to alarms signifying rapidly falling glucose: “The [accuracy] is not always reliable when bloods are falling quickly but it is invaluable at showing trends and pre-empting hypo.” There is no recommended frequency that denotes a good level of engagement or awareness of glucose trends; however, regular review of CGM readings is important, as responding to alarms alone will allow minimal appreciation of glucose trends.

Fear of hypoglycemia is a serious concern for children vulnerable to hypoglycemia and their parents, negatively impacting their quality of life (36). All respondent groups felt strongly that physical activity is safer for children when wearing CGM, highlighting that families find CGM a supportive tool in monitoring blood glucose levels during exercise for children with CHI. On all quality of life measures, families agreed that CGM had a positive impact, supporting previous findings (37). Free text comments highlighted that “the peace of mind from having it on is amazing” and that through continual education, CGM has enabled parents to feel empowered about transition to adult care: “We can plan for a future when my child can take over responsibility of his own care.” Future research is needed to understand whether CGM use influences uptake of early socialization opportunities, eg, sports clubs and Early Years settings, and whether there is an impact on parental well-being and employment.

Interestingly, parents in our study (who had been using CGM for a median of 1 year 3 months) reported improved sleep with CGM; only 9% reported increased need for SMBG overnight, whereas other studies have reported worsened sleep and high rates of CGM discontinuation (23). The impact of CGM on both carer and patient sleep quality likely influences the desire to use CGM; therefore, more research is needed to understand whether there are baseline differences between those families who report sleep improvements and those who report sleep deterioration with CGM use.

Parents expressed frustration at devices’ limited range and having to ensure their child carries a dedicated CGM receiver or a smartphone that must remain within range of the CGM (“If the CGM could link to a smaller device such a watch, that would make her life easier”). Recent advancements in CGM technology, including enhancements in Bluetooth connectivity and the introduction of direct-to-watch capabilities, have improved CGM range and usability. Further research is needed to understand the proportion of families utilizing smart devices, such as watches, to optimize CGM use and whether these advancements have addressed challenges described by parents.

Current barriers to use of CGM for CHI include a lack of evidence that CGM use reduces hypoglycemia, concerns around device accuracy and sensitivity, and the financial implications of provision of CGM consumables (11, 12). Future research must establish the impact of CGM on the long-term management of CHI patients, including severity of hypoglycemia, use of emergency rescue medication, emergency hospital admissions, and neurodevelopmental outcomes.

## Conclusion

Our data demonstrate that, for a significant number of patients with CHI, CGM has the capacity to improve quality of life while supporting CHI management across all areas measured. As CGM technology develops, improvements to accuracy and sensitivity in the hypoglycemic range, alongside device size, application, and range, should all be prioritized by manufacturers. Improvements in patient and caregiver satisfaction are important goals of management and should be considered alongside biochemical markers and blood glucose trends. It is essential that clinicians are mindful of patient-reported outcomes (38) when acting as gatekeepers to new technology.

## Data Availability

Restrictions apply to the availability of some or all data generated or analyzed during this study to preserve patient confidentiality. The corresponding author will on request detail the restrictions and any conditions under which access to some data may be provided.
